# Synchronization of optically self-assembled nanorotors

**DOI:** 10.1126/sciadv.adn3485

**Published:** 2024-03-08

**Authors:** Ximin Cui, Vasilii Mylnikov, Peter Johansson, Mikael Käll

**Affiliations:** ^1^ Department of Physics, Chalmers University of Technology, 412 96 Göteborg, Sweden.; ^2^ College of Electronics and Information Engineering, Shenzhen University, Shenzhen 518060, China.; ^3^ School of Science and Technology, Örebro University, 701 82 Örebro, Sweden.

## Abstract

Self-assembly of nanoparticles by means of interparticle optical forces provides a compelling approach toward contact-free organization and manipulation of nanoscale entities. However, exploration of the rotational degrees of freedom in this process has remained limited, primarily because of the predominant focus on spherical nanoparticles, for which individual particle orientation cannot be determined. Here, we show that gold nanorods, which self-assemble in water under the influence of circularly polarized light, exhibit synchronized rotational motion at kilohertz frequencies. The synchronization is caused by strong optical interactions and occurs despite the presence of thermal diffusion. Our findings elucidate the intricate dynamics arising from the transfer of photon spin angular momentum to optically bound matter and hold promise for advancing the emerging field of light-driven nanomachinery.

## Teaser

Gold nanorods operated as laser-driven rotary nanomotors in water synchronize their movement while spinning at kilohertz rates.

## INTRODUCTION

Beginning with its discovery in coupled pendulum systems by Huygens (*1*), synchronization of oscillatory phenomena has been observed across diverse domains of science, including physics (*2*), biology (*3*), chemistry (*4*), and even human social activities (*5*), and spanning from cosmic to atomic scales. In daily life, synchronization is used in, for example, signal processing (*6*), clock calibration (*7*), data communication (*8*), and navigation (*9*). Broadly defined, synchronization entails the phase locking of oscillatory rhythms among two or more interacting entities (*10*). For this to occur, the interaction between oscillators needs to be of such nature and strength that mutual feedback eventually forces the individual oscillators to adjust their rhythms into synchrony. Achieving this for mechanical oscillators at the nanoscale is challenging not only because of the difficulty of constructing and precisely assembling tiny dynamical systems of oscillators but also because thermal diffusion, which is unavoidable at the nanoscale, will tend to randomize oscillator motion, thereby causing dephasing. Nevertheless, here, we show that a high degree of synchronization is possible for driven nanoscale rotary oscillators (“nanorotors”) interacting through a strongly dissipating aqueous environment. In contrast to recent studies of hydrodynamic synchronization of magnetic particle systems (*11*, *12*), the nanorotors operate at kilohertz frequencies, and they are driven by and couple through light.

When a laser beam strikes a small particle, both total energy and total momentum need to be conserved in the light-matter interaction. Conservation of linear momentum implies that light scattering and absorption cause recoil forces acting on the particle. The particle can be pushed forward (“radiation pressure”) or even be trapped at a single location if the beam is tightly focused (“laser tweezing”) (*13*, *14*). If several particles reside within the same laser field, recoil forces appear not only because of the primary optical interaction but also because of coherent interparticle scattering. The result is that particles tend to self-organize (“optically bind”) (*15*) a wavelength apart into ordered arrays (“optical matter”) (*16*). Moreover, conservation of angular momentum results in torques acting on the particle (*17*, *18*). This happens, for example, if the laser beam is circularly polarized, but the scattering is mainly linearly polarized, as is the case when the particle is elongated in the plane of polarization (*19*). The particle will then rotate at a frequency determined by the applied laser power and the rotational drag and in a direction given by the handedness of light.

Research and applications of optical manipulation in the microdomain have been rife for decades, but it is only in the past several years that it has become possible to bring the insights gained to the nanoscale. One of the main drivers behind this development is the increasing availability of single-crystal gold and silver nanoparticles. By virtue of localized surface plasmon resonance effects, these particles can experience strongly enhanced optical forces and torques, thus enabling studies of smaller structures and new phenomena (*19*–*21*). One prominent example concerns optical binding and optical matter formation in weakly focused and circularly polarized laser light. Multiple plasmonic nanoparticles subject to these conditions spontaneously self-organize into various nonequilibrium two-dimensional (2D) configurations (*22*, *23*), revealing intriguing phenomena, such as negative optical torque (*24*–*26*) and self-healing effects (*27*), originating in the mutually scattered light between neighboring nanoparticles. However, these works were based on the self-assembly of spherical nanoparticles, for which individual particle orientations cannot be determined.

In this work, we instead study optical binding of elongated nanoparticles in the form of plasmonic gold nanorods (Au NRs). Because of their pronounced optical anisotropy, these NRs can be made to rotate at tens of kilohertz in water when trapped by a focused circularly polarized laser beam, making the particles act as efficient rotary nanomotors (*28*, *29*). We use stroboscopic imaging and stochastic simulations to reveal and analyze synchronization in small clusters of optically bound and rapidly spinning nanorotors. Rotational synchronization through optical interaction has only been previously observed for slowly moving microparticles (*30*–*32*) and theoretically predicted for high aspect ratio dielectric nanowires (*33*).

## RESULTS

### Self-organization of optical matter arrays

**Fig. 1.**
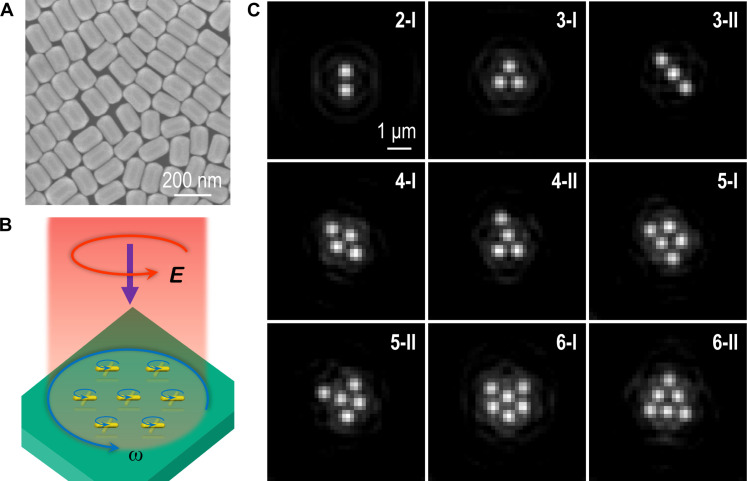
Light-induced self-assembly of Au NRs into optical matter arrays. (**A**) Scanning electron microscopy image of the Au NRs with an average length of 166 ± 7 nm and a diameter of 101 ± 6 nm. (**B**) Schematic of rotation of a Au NR optical matter array illuminated by a circularly polarized laser beam, which provides optical angular momentum that forces the NRs to spin around their own axis and orbit around their common center of mass. (**C**) Optical micrographs of representative optical matter arrays consisting of two to six Au NRs.

We investigate Au NRs with a length of 166 ± 7 nm and a width of 101 ± 6 nm synthesized by a modified seed-mediated method (see Materials and Methods and Fig. 1A) (*34*). The NRs support two dominant dipolar localized surface plasmon resonance modes, located at ~570 nm (short axis, transverse resonance) and ~800 nm (long axis, longitudinal resonance), respectively (fig. S1). A droplet of the NR solution is inserted in a thin liquid sample cell and observed under continuous white-light dark-field (DF) illumination in an inverted microscope (see Materials and Methods and fig. S2). Analogous to spherical particles used in previous works (*25*, *35*), the NRs are found to self-organize into 2D optical matter arrays when subject to a weakly focused and circularly polarized laser beam of sufficient power (Fig. 1B). This process involves the three optical force effects mentioned above: radiation pressure, which pushes the NRs against the upper cover glass of the sample cell; the optical gradient force, which attracts NRs into the Gaussian laser spot [full width at half maximum (FWHM), ~2.5 μm; fig. S3]; and optical binding (*15*), which drives optical matter formation.

We observe various optical matter isomers (Fig. 1C), all with characteristic nearest-neighbor distances approximately given by the laser wavelength in water, λ = 1064/1.33 = 800 nm, which is the expected optical binding distance. Random transitions between different close-packed hexagonal structures occasionally occur because of translational Brownian diffusion (movies S1 to S3). The optical matter arrays slowly orbit as rigid bodies around their common center of mass due to transfer of angular momentum from the circularly polarized trapping field (movies S4 to S7). The sense of rotation is the same as the input polarization and the orbital rotation frequency increases approximately linearly with the number of particles in the cluster (fig. S4). All in all, the behavior of the NRs appears very similar to optical matter arrays formed by plasmonic nanospheres trapped under similar conditions (*25*, *35*, *36*). However, this resemblance is partly superficial and caused by the difficulty of resolving the NR spinning motion using standard DF observation.

### Particle dynamics in the absence of Brownian diffusion

To visualize how this rapid NR spinning influences and is affected by optical binding, we discretized and iteratively solved the equations of motion for rotation and translation in systems comprising interacting identical NRs in a uniform aqueous environment. Inertial effects and hydrodynamic interactions are negligible for the particle sizes and interparticle distances considered here (see discussion in the Supplementary Materials). The NR spinning dynamics is then governed by the equation of motion $\dot{\varphi }=\left(M_{\text{opt}}+M_{s}\right)/\gamma_{\text{rot}}$ , where $\dot{\varphi }$ is the instantaneous NR angular velocity, *M*_opt_ is the optical torque, *M*_s_ is a stochastic torque, causing rotational Brownian motion, and γ_rot_ is the rotational friction coefficient. The average rotation frequency in a uniform circularly polarized field is thus *f*_0_ = *M*_opt_/(2πγ_rot_) since the time average of *M*_s_ vanishes. Analogous equations of motion can be formulated for translation parallel and perpendicular to the NR long axis.

**Fig. 2.**
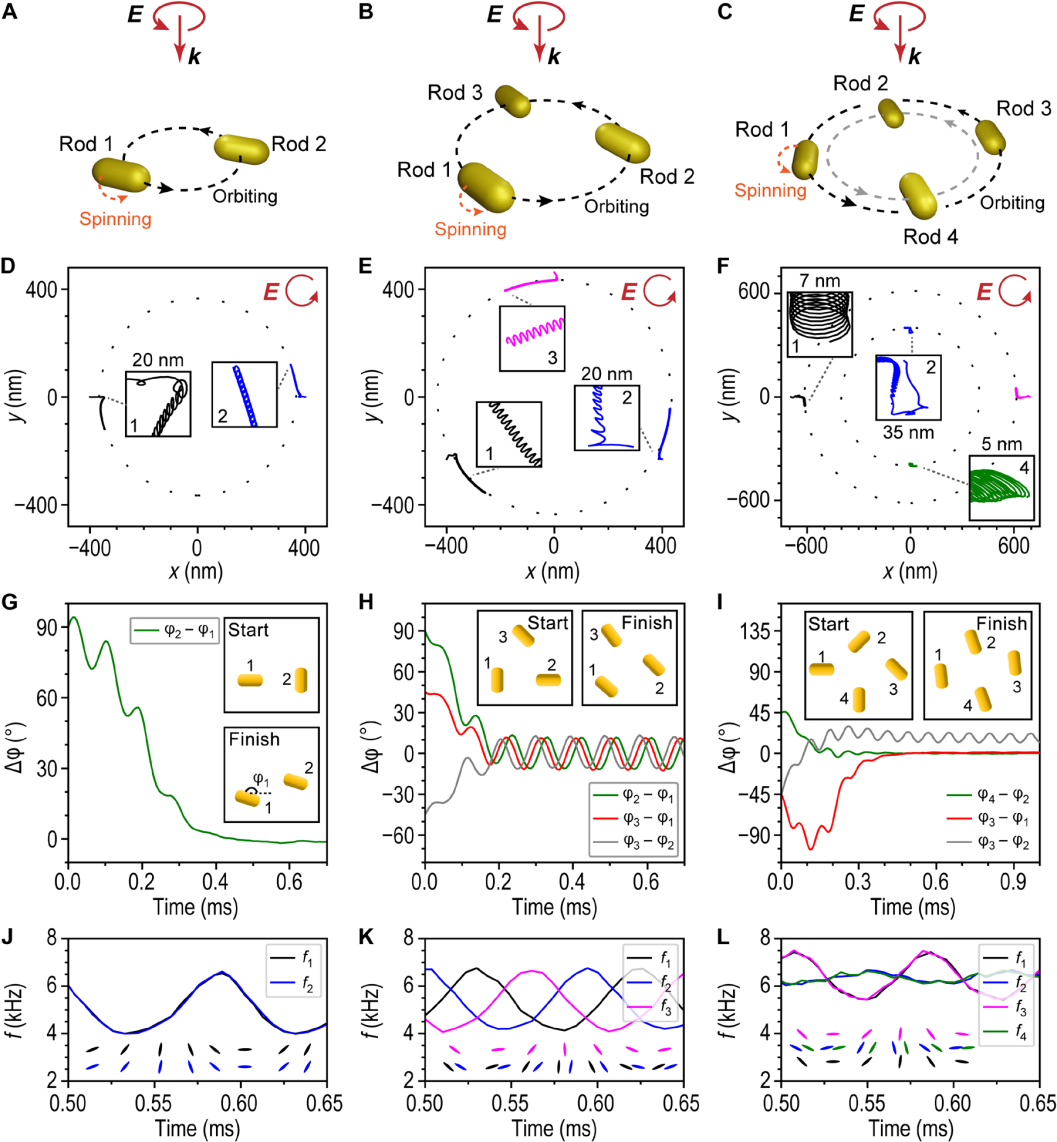
Dynamic simulations of rotational synchronization and alignment of optically bound Au NRs. (**A** to **C**) Schematic illustrations of the investigated NR configurations. (**D** to **F**) Evolution of the NRs’ centers of mass. (**G** to **I**) Evolution of the NRs’ relative angular orientation, with φ*_i_* being the angle between the long axis of NR *i* and the *x* axis. Insets indicate initial and representative dynamic equilibrium orientations. (**J** to **L**) Variation in NR spinning frequency at dynamic equilibrium together with instantaneous particle configurations at specific time points. Simulations were performed for a circularly polarized incident beam with Gaussian intensity distribution (λ_0_ = 1064 nm, FWHM = 2.5 μm, and *P* = 430 mW for dimers and trimers and *P* = 530 mW for tetramers).

In a first simulation study, we used a finite-difference time-domain (FDTD) model to accurately describe the NR optical response in a beam with a Gaussian intensity distribution similar to the experiment. The optical forces and torques were calculated from the Maxwell stress tensor, while the friction coefficients for translation and rotation were obtained from a model (*37*) describing the diffusion of prolate spheroids (fig. S5). Simulations were performed for two, three, and four interacting NRs restricted to move in the *xy* plane (Fig. 2, A to C). At this stage, we deliberately neglected the stochastic force components to isolate the effects of optical forces. Starting from closely packed configurations with nearest-neighbor interparticle spacing *d* = 800 nm, we tracked the NRs’ centers of mass and the angles φ between the NRs’ long axes and the *x* axis as a function of time (Fig. 2, D to F and G to I).

For the simplest case of an NR dimer (Fig. 2A), the simulation starts with the NRs oriented perpendicularly to each other. Within a fraction of a millisecond, the particles relax toward the beam center, and the relative particle orientation evolves from perpendicular to parallel (∣φ_2_ − φ_1_∣ = 0; Fig. 2G). The NR dimer then begins to orbit in the same direction as the handedness of the incident polarization with an average orbital rotation frequency of $\bar{\Omega }\approx 7.1$ Hz (Fig. 2D), which agrees reasonably well with experimental results (fig. S4). We interpret the smaller average particle separation at dynamic equilibrium, $\bar{d}\approx 730$ nm, as due to the competition between optical binding, which strives to keep the particles ~800 nm apart, and the gradient force, which pulls them toward the beam center.

The coupling between NR spinning and optical binding manifests itself through anharmonic oscillations around dynamic equilibrium. The most notable effect is a large fluctuation in the spinning frequency of $\text{∆}f/\bar{f}\approx \pm 24\%$ around the average value of $\bar{f}\approx 5.3\;\text{kHz}$ (Fig. 2J), but the coupling is also evident from the peculiar spiral tracks of the NRs’ centers of mass visible in Fig. 2D. Results for the trimer case (Fig. 2, E, H, and K) are qualitatively similar to the dimer but with slightly different dynamic equilibrium averages ( $\bar{d}\approx 750$ nm, $\bar{\Omega }\approx 8.5$ Hz, $\bar{f}\approx 5.3\;\text{kHz}$, and $\text{∆}f/\bar{f}\approx \pm 24\%$ ). However, the NRs are no longer perfectly aligned but exhibit a small oscillation in relative orientation of around ±12° (Fig. 2H). The diamond-shaped tetramer (Fig. 2, F, I, and L) contains two inequivalent pairs of particles that orbit at different radii from the beam center. The local intensity at these radii differs by ~10%, but the NRs still spin with the same frequency ( $\bar{f}\approx 6.3\;\text{kHz}$), although with a relative phase shift that fluctuates around ~16°.

The angular synchronization and spinning frequency oscillations seen in Fig. 2 can be qualitatively understood from a dipole interaction analysis. For the simplest case of two identical and optically bound NRs that are only polarizable along their long axis and that spin because of a right-handed incident field, approximate expressions for the instantaneous frequencies read (see the Supplementary Materials for a derivation):

$$\begin{array}{c} f_{1}=f_{0}+∆f[\text{sin}(2\varphi_{1})+\text{sin}(2\varphi_{2}-2\varphi_{1})]\\ f_{2}=f_{0}+∆f[\text{sin}(2\varphi_{2})-\text{sin}(2\varphi_{2}-2\varphi_{1})] \end{array}$$ (1)

Here, $f_{0}={\dot{\varphi }}_{0}/2\pi$ is the spinning frequency in the absence of optical binding, ∆*f* is determined by the coupling strength, set by the particle polarizability and the interparticle distance, and φ_1,2_ are the angles between the NRs long axes and the dimer axis. The factor of 2 in the arguments reflects the inversion symmetry of the NRs.

First, consider the case when the NRs are parallel and already in phase, such that the second term in parenthesis vanishes. Coupling then enters through the ∆*f* sin (2φ) terms, which can be interpreted as due to an average aligning torque striving to orient the dipoles orthogonally to the dimer axis, for which the optical binding interaction is strongest. Consequently, the NRs accelerate (decelerate) when their long axes approach (have passed) the orthogonal orientation, resulting in maximum (minimum) angular velocities at φ_1,2_ ≈ 45^°^ (135^°^) in good agreement with Fig. 2J.

Next, consider the case when φ_2_ − φ_1_ ≠ 0. If φ_2_ > φ_1_ (NR1 lags behind NR2), then the second term in parenthesis will add to the velocity of NR1 but subtract from the velocity of NR2 (and vice versa for φ_2_ < φ_1_). Thus, NR1 will eventually align with NR2, as seen in the simulations. We note that the coupling between the different angles is identical to the one in the Kuramoto model, which can be considered the master equation of synchronization, and that in its various extensions allows for a distribution of natural frequencies for the different oscillators and thermal noise (*38*).

Notice that the average spinning frequencies obtained from Eq. 1 equal the uncoupled frequency (*f*_0_), implying that, in this first approximation, optical binding does not affect the time-averaged optical torque extracted from the incident field (*M*_opt_). However, conservation of angular momentum means that the incident torque must be shared between a spin torque (*M*_spin_), driving the NR spinning motion, and an orbital torque (*M*_orb_), causing the orbital motion, such that *M*_opt_ = *M*_spin_ + *M*_orb_. The orbital torque is not included in Eq. 1, but we can estimate its relative magnitude as $M_{\text{orb}}/M_{\text{spin}}\approx \bar{\Omega }r^{2}{\bar{\gamma }}_{t}/\bar{f}\gamma_{\text{rot}}$ , where *r* is the radius of the NR orbit and ${\bar{\gamma }}_{t}$ is the average translational friction coefficient. Inserting values from Fig. 2 yields that *M*_orb_/*M*_spin_ is of the order of a few percentage, indicating that the orbital motion has little influence on the overall synchronization process.

### Statistical evidence of NR rotation synchronization in optical matter arrays

Although the NR rotation synchronization behavior seen in the FDTD simulations is a natural consequence of the completely deterministic equations of motion and the symmetries of the studied systems, it is not obvious that the effect can be observed experimentally and, to what extent, it survives in the presence of Brownian motion. To examine this, we implemented two methods that provide compelling statistical evidence of synchronization: stroboscopic imaging and dynamic simulations of particle trajectories including rotational and translational diffusion.

In the experimental realization, we used the anisotropic NR scattering intensity as a proxy for angular orientation. By inserting a polarizer in front of the complementary metal-oxide semiconductor (CMOS) camera detector and by replacing the white-light source in the DF microscope by a light-emitting diode (LED) with unpolarized emission and spectral profile similar to the longitudinal plasmon band, an NR appears bright (*I*_max_) when its long axis is parallel to the polarizer and faint (*I*_min_) when it is perpendicular. FDTD simulations aimed to mimic the experimental conditions yield *I*_max_/*I*_min_ ≈ 5 (fig. S7). To resolve the NR spinning motion, we run the LED in stroboscopic mode using a flash time of *T*_f_ = 100 μs, which gives a reasonable trade-off between temporal resolution and signal amplitude. The time interval between flashes was ~1.6 ms and matched the frame rate of the camera, such that each image is exposed to one flash only (fig. S2).

**Fig. 3.**
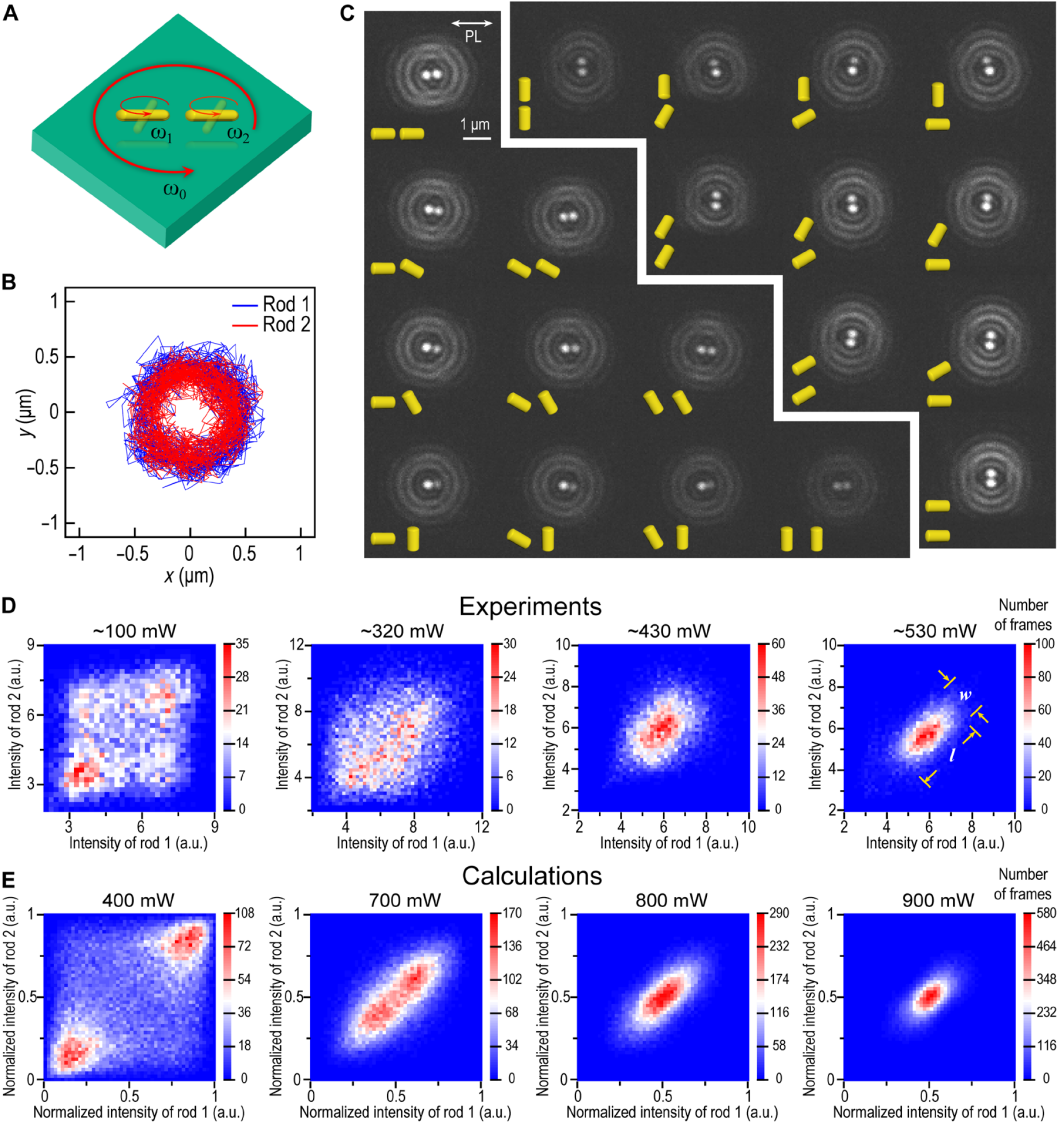
Statistical analyses of optically bound and spinning NR dimers. (**A**) Schematic of an optically bound and spinning NR dimer. Experiments are performed on pairs of Au NRs trapped in 2D using a circularly polarized laser beam (λ_0_= 1064 nm and FWHM = 2.5 μm). NR dimers orbit around the optical axis, while the particles spin around their centers of mass. (**B**) The orbital motion can be tracked using standard white-light DF microscopy. (**C**) DF microscopy using stroboscopic red-light illumination combined with polarization-filtered imaging reveals intensity fluctuations due to variations in NR orientation with the respect to the polarizer. The figure shows various snapshots for cases when the dimer axis is nearly parallel and nearly perpendicular to the polarizer orientation, indicated in the top left corner, together with schematics indicating possible NR orientations compatible with the recorded scattering intensities. (**D**) Scatter plots of intensities *I*_1_ versus *I*_2_, extracted from >10,000 separate images and binned into heatmaps, for four different laser powers. The intensities concentrate along the diagonal, *I*_1_ = *I*_2_, as expected for synchronized spinning of aligned NRs. (**E**) Dynamic simulations of optically bound Au prolate spheroids based on extended Mie theory and including rotational and translational diffusion in 2D. The laser powers used in the simulations were chosen to produce heatmaps similar to those observed experimentally. The corresponding average NR spinning frequencies ( $\bar{f}$ ) and order parameters (*r*) are 1.9 kHz/0.81 (400 mW), 3.3 kHz/0.89 (700 mW), 3.7 kHz/0.90 (800 mW), and 4.2 kHz/0.92 (900 mW), respectively. a.u., arbitrary units.

The optically bound NR dimer is expected to orbit around the optical axis in the same direction as the incident polarization (Fig. 3, A and B), which agrees well with simulation results (Fig. 2D). Figure 3C gives examples of images acquired using stroboscopic illumination for this system. It is obvious that the intensities of the two particles fluctuate from frame to frame, indicating their varying angle φ with respect to the polarizer orientation. Possible NR configurations compatible with the intensity variations are shown schematically in the corresponding insets. The distribution in recorded intensities of the individual particles varies with applied laser power (fig. S8). Whereas the high-power distribution can be fitted well with a Gaussian, the low-power distribution instead shows distinct peaks at high and low intensities. This difference can be qualitatively understood by considering the expected variation in scattering intensity with angle φ between the NR long axis and the polarizer, *I*(φ) = *I*_max_cos^2^φ + *I*_min_sin^2^φ, and the variation in spinning frequency with applied power, $\bar{f}\propto P$ . In the limiting case of $T_{f}\bar{f}\to 0$ , that is, when the angular variation during the stroboscopic flash is negligible, *I*(φ) can be analytically inverted to yield a probability density ${𝒫}^{0}\left(I\right)\propto 1/\sqrt{\left(I_{\text{max}}-I\right)\left(I-I_{\text{min}}\right)}$ of recording a certain scattering intensity. This function exhibits square root cusps at *I*_max_ and *I*_min_, which, when broadened by Brownian diffusion and experimental noise, produce peaks at high and low intensities. In the opposite limit, $T_{f}\bar{f}\to \infty$ , that is, when the spinning motion is so fast that an NR samples all angles with equal probability during the flash time, the distribution will instead be a Gaussian peaked at the average intensity (*I*_max_ + *I*_min_)/2.

We next examine the correlation between the two intensities recorded for optically bound NR dimers measured at increasing laser power (Fig. 3, D and E, and see fig. S9 for corresponding results on dimers of spherical Au nanoparticles). Unfortunately, since the particle positions changes between flashes due to translational diffusion, including sudden jumps, it is not possible to trace the intensity fluctuations of a specific particle over time with certainty. Instead, we arbitrarily assign the particle with the highest (lowest) frame coordinate as NR1 (NR2) and record their respective intensities (*I*_1_, *I*_2_) for a large number of frames (*N* > 10,000). Scatter plots of *I*_1_ versus *I*_2_ for all frames then provide a measure of the joint probability distribution 𝒫(*I*_1_, *I*_2_) and thereby a statistical insight into the relative particle orientation and degree of synchronization. The data in Fig. 3D show that the data points gather around the diagonal *I*_1_ = *I*_2_. Such a positive covariance can only occur if the NRs are, on average, aligned and rotate in synchrony in the same direction as in the dynamic FDTD simulations (the possibility of synchronized rotation in opposite directions can be excluded, given the handedness of the driving field). These data constitute the primary experimental evidence for rotational synchronization.

To simulate the corresponding intensity distributions, one needs to solve the equations of motion in the presence of rotational and translational Brownian diffusion using a time step that is substantially smaller than the characteristic diffusion times and over long enough trajectories to obtain statistically robust results. This is not practically achievable using the FDTD approach discussed in the previous section. We therefore developed an efficient code based on the T-matrix and extended Mie theory for electromagnetically coupled prolate spheroids with shape and size similar to the NRs used in the experiments (fig. S5). The method is briefly described in the Supplementary Materials and allows us to iterate the full equations of motion, including the stochastic force components, over several seconds with a time step of δ*t* = 2 μs. These data can then be averaged over time intervals *T*_f_ = 100 μs as a comparison to the experimental results.

Figure 3E shows simulation results for increasing applied power chosen to produce scatter plots that resemble the experimental data in terms of the ratio between the width and length (*w*/*l*) of the distribution along the diagonal *I*_1_ = *I*_2_. For low power, the intensity points cluster around the opposite ends of the diagonal because of the square root cusps at high and low intensities mentioned above, whereas high power produces Gaussian distributions. However, the distributions are always stretched out along the diagonal, meaning that synchronization occurs at all power levels. We can now estimate the average NR spinning frequency for the different laser powers used in the experiment. We find that $\bar{f}$ increases linearly with applied power, as expected, to reach ~4.2 kHz at the highest power used. We can also estimate the degree of synchronization by defining a time-averaged order parameter $r=N^{-1}\langle |\sum_{p=1}^{N}\;\text{exp}[2i\varphi_{p}(t)]|\rangle$ , where *N* is the number of particles and *r* = 1 (0) implies perfect (random) phase synchronization. We find that *r* varies little, from ~0.81 to ~0.92, within the investigated power range, which implies that the main cause behind the change in scatter plot appearance with increasing power is the increasing angular range covered during a flash. By gradually decreasing the simulated flash time, we find that the intensity variation perpendicular to the heatmap diagonal shifts from a Gaussian to a cusp-shaped curve as $T_{f}\bar{f}\to 0$ (fig. S10), which is the expected result since synchronization is only limited by thermal fluctuations. The simulations also allow us to verify that the randomization of particle identity performed in the experimental analysis does not give rise to false-positive covariances, even for cases when the scattering amplitudes of the individual NRs vary substantially (fig. S11).

**Fig. 4.**
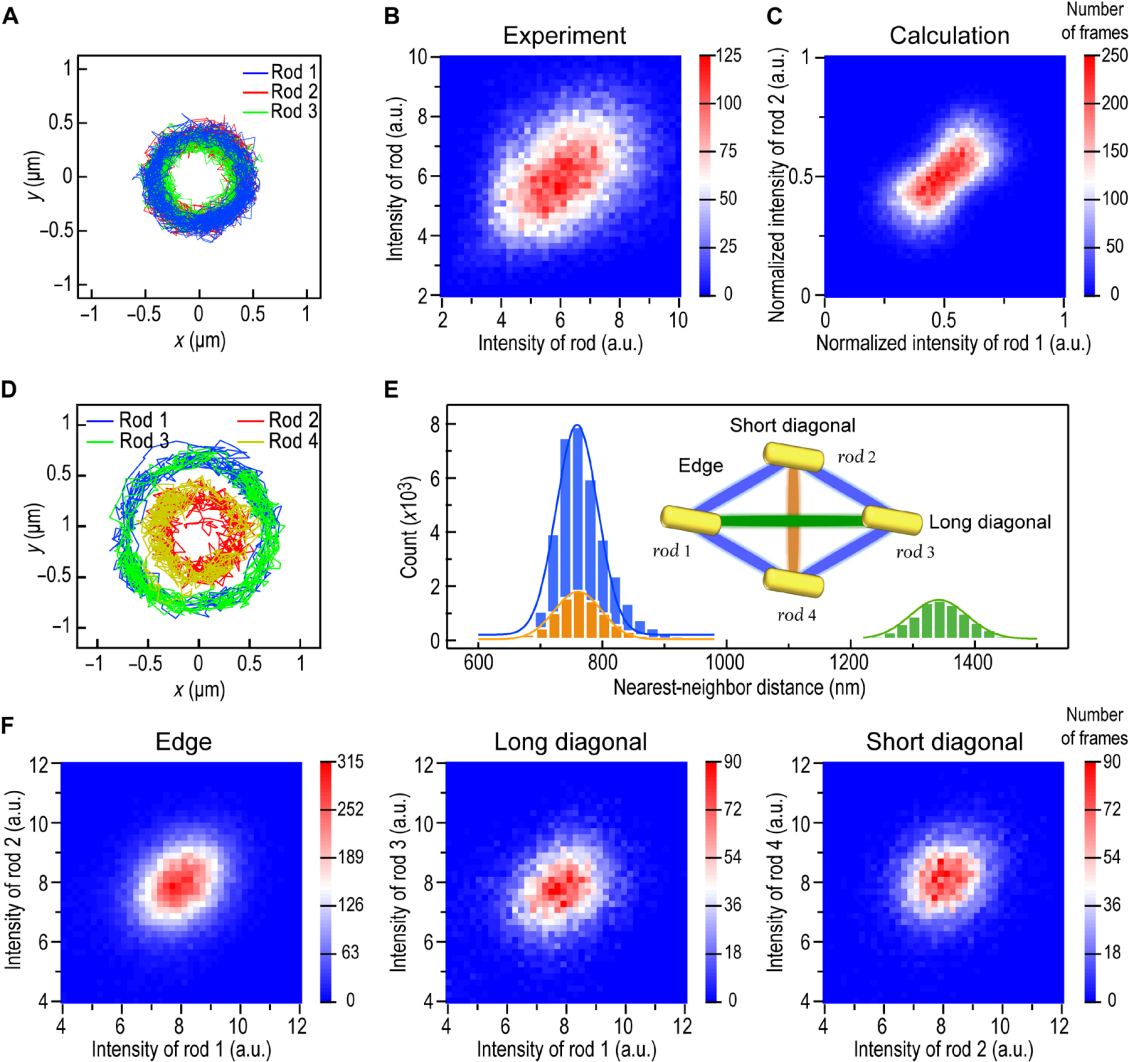
Statistical analysis of optically bound NR trimers and tetramers. (**A**) Individual particle trajectories of three NRs, optically bound into a trimer with triangular symmetry, recorded using white-light DF microscopy. (**B**) Binned scatter plot of intensity correlations of two particles in a trimer recorded using stroboscopic illumination. (**C**) Simulated intensity correlations of Au prolate spheroids optically bound into a trimer at 800 mW, resulting in an average spinning frequency of 3.8 kHz and an order parameter of 0.92. (**D**) Particle trajectories of four NRs optically bound into a diamond-shaped tetramer recorded using white-light DF microscopy. (**E**) Histograms of center-to-center particle distances obtained from the data in (D). Solid curves are Gaussian fits. Inset indicates the NR pairs at the edge and across the long and short diagonals. (**F**) Binned scatter plots of intensity correlations for a diamond-shaped tetramer recorded using stroboscopic illumination. *I*_1_ versus *I*_2_ refer to correlations between neighboring NRs at the edge of the tetramer, while *I*_1_ versus *I*_3_ and *I*_2_ versus *I*_4_ refer to correlations across the long and short diagonals, respectively. The stroboscopic data for the trimer and tetramer were recorded using incident laser powers of ~430 and ~530 mW, respectively, and were collected over >10,000 separate DF images each.

Figure 4 summarizes results for a trimer and a tetramer (additional data in figs. S12 and S13). The trimer is optically bound in a close-packed triangular configuration with a side length of ~760 nm (Fig. 4A), similar to the dimer case, and slowly orbits around the optical axis with a rotation frequency of ~1.5 Hz (fig. S4), which is in reasonable agreement with FDTD simulation results for the same applied power (*P* ≈ 430 mW). DF images using stroboscopic illumination reveal a variety of instantaneous particle intensities and orientational configurations (fig. S12A). However, when a large number of images are analyzed in the same way as for the dimer, and the pairwise intensity correlations are plotted as a heatmap of occurrences (Fig. 4B), one finds a clear positive covariance, demonstrating NR alignment and synchronization also in this case. The shape of the distribution is almost identical to that of a dimer recorded for the same applied power (*w*/*l* ≈ 0.59). Note that the three pairwise intensity correlations in the trimer are statistically identical since the individual particles cannot be tracked in the stroboscopic measurements (fig. S12, B and C). Dynamic simulations of three interacting and diffusing prolate spheroids based on Mie theory also produce results analogous to the dimer case (Fig. 4C). The average NR spinning frequency is found to be ~3.8 kHz for an applied laser power that produce a scatter plot similar to the experiment, and the corresponding order parameter is then *r* = 0.92.

The tetramer analyzed in Fig. 4 is optically bound in a closely packed diamond configuration, with a short axis of ~760 nm and a long axis of ~$\sqrt{3}\cdot 760\;\text{nm}$ , and it orbits at ~2.3 Hz (Fig. 4D). There are now three distinct and resolvable pair correlations (Fig. 4E). All three show similar positive covariances (Fig. 4F), but the shape of the distributions is clearly more circular (*w*/*l* ≈ 0.78), indicating a lower degree of synchronization. The dynamic Mie simulations showed synchronized spinning in this case as well, but the four particles converged to a square arrangement in contrast to the FDTD result (Fig. 2F), while a diamond was only found for plane wave incidence in the dynamic Mie simulations, indicating that the structure is highly sensitive to the optical gradient forces induced by the Gaussian beam.

## DISCUSSION

In summary, we have demonstrated that optically bound plasmonic NRs that are driven to spin at several kilohertz are able to synchronize their rotary motion in a strongly dissipative aqueous environment. The degree of synchronization is unexpectedly high (order parameter, ~0.8 to 0.9) and is largely independent of the spinning frequency within the investigated range of incident laser powers.

Previous investigations of optically bound clusters of plasmonic nanospheres have highlighted orbital rotation phenomena and the possibility of using this motion for self-assembled rotary “optical matter machines” (*23*). These structures could, in principle, be used to probe or exert torque on the surrounding environment, similar to solid nanofabricated microstructures (*39*, *40*). However, in the small NR systems investigated here, we find that the incident angular momentum is predominantly spent on spinning the particles around their own axis rather than driving orbital motion, and this is likely to be the case also for small systems of nanospheres. In consequence, optical matter machines based on a small number of optically bound particles will be rather inefficient. It is an open question to what extent optical binding in even larger optical matter arrays could decrease or even quench the spinning motion, thereby maximizing the orbital torque such a system could produce.

Studies of optically bound and rotating NRs could be extended to systems that are more complicated than the cases investigated here. For example, by including NRs of varying aspect ratio within the same optically bound cluster, it should be possible to study to what extent synchronization survives in the presence of rotation frequency disorder (*38*). Further, by using elliptical rather than circular polarization, it might be possible to study synchronization in the presence of an additional aligning potential, which, in the case of single NRs, leads to discrete stochastic jumps rather than continuous rotation (*41*). Moreover, rapid progress in levitated optomechanics over the past several years indicates that rotational synchronization might be observable under close-to-vacuum conditions, for which viscous damping is very weak (*42*, *43*). A recent example in this direction used two polystyrene microbeads trapped next to each other in separate standing wave patterns, which allowed for the demonstration of coherent orbital motion and synchronization due to hydrodynamic and optical interactions (*32*). Experiments on plasmonic NRs are not feasible in a vacuum due to photothermal heating effects, but vacuum trapping and rotation of NR-like clusters (dumbbells) of dielectric nanoparticles have been demonstrated (*44*, *45*). It will be challenging to gather data on the rotational interactions of these structures due to the megahertz-gigahertz frequencies encountered, but it might be possible using stroboscopic illumination with ultrashort laser pulses. By confining two or more dielectric NRs in separate vacuum traps with controlled polarization ellipticity and optical phase, it would be possible to study a wide range of rotational-vibrational coupling effects, perhaps even down to the quantum limit (*46*).

## MATERIALS AND METHODS

### Sample preparation

Colloidal Au NRs were synthesized using a previously reported seed-mediated method (*34*). The particles were stabilized with a binary surfactant mixture consisting of hexadecyltrimethylammonium bromide (CTAB) and sodium oleate in aqueous solution. The seed solution was prepared by rapidly adding a freshly prepared ice-cold aqueous NaBH_4_ solution (6 mM, 1 ml) into a mixture of CTAB (0.1 M, 10 ml) and HAuCl_4_ (0.01 M, 0.25 ml) under vigorous stirring (1200 rpm). After 2 min of stirring, the seed solution was aged for >30 min at room temperature before further use. A second solution containing CTAB (9 g) and sodium oleate (1.543 g) dissolved in warm water (50°C, 250 ml) was prepared. After cooling to 30°C, AgNO_3_ (4 mM, 12 ml) and HAuCl_4_ (1 mM, 250 ml) were added gradually under mild stirring (700 rpm), resulting in a colorless solution after ~90 min. HCl (37 weight %, 1.5 ml) was injected, and the solution was stirred at 400 rpm for 15 min, after which ascorbic acid (0.1 M, 0.8 ml) was added under vigorous stirring for 30 s, followed by adding 10 μl of the seed solution. The resultant mixture was stirred for 30 s and then kept undisturbed at room temperature overnight. The resulting Au NR sample was washed and concentrated into water for further use. The size and morphology of the NRs were determined by a scanning electron microscope (Leo Ultra 55 FEG SEM, Carl Zeiss).

### Optical experiments

The optical system (fig. S2) was constructed around an inverted microscope (Nikon Eclipse Ti) equipped for DF observation and optical tweezing. A 10-μl droplet of the diluted colloidal NR solution was placed in a 100-μm-thin sample chamber created between two glass slides. The output of a 1064-nm continuous wave laser (Cobolt Rumba 2W) was adjusted in power, beam width, and polarization and then focused on the back focal plane of a 40× objective (Nikon CFI Plan Apo Lambda 40×, numerical aperture = 0.95) to yield a circularly polarized Gaussian spot with an FWHM of ~2.5 μm (fig. S3) at the upper interface of the sample chamber. DF scattering from the NRs, excited by either a white-light lamp or a narrow-band red LED (Thorlabs Solis-740 nm, bandwidth of 45 nm, 3.3 W), was captured by a CMOS camera (Andor Neo 5.5 sCMOS). A notch filter (Edmund Optics 1064 nm, OD 6) was used to block laser reflections and laser back-scattering from the NRs. Stroboscopic observation of NR rotation and synchronization was performed with a linear polarizer inserted in front of the camera. The red LED driver was set to a flash time of ~100 μs and a flash frequency of 595 Hz, while the CMOS camera was operated at a frame rate of 633 Hz by reducing the readout frame size to 60 × 60 pixels. This guarantees that not more than one illumination pulse contributes to each captured image. Determination of NR positions and brightness was carried out using TrackMate in ImageJ (*47*).

### Numerical simulations

Electrodynamic simulations of optical properties were performed using Ansys Lumerical FDTD, in which an NR was described as a truncated cylinder with spheroidal caps and using extended Mie theory, in which an NR is described as a prolate spheroid (fig. S5). Optical forces and torques were obtained from the Maxwell stress tensor, using a circularly polarized beam with Gaussian intensity distribution and an FWHM of ~2.5 μm as incident light. The equations of motion for translation and rotation in a fixed plane normal to the trapping incidence direction were discretized using the Euler method. We neglected inertial effects and hydrodynamic interparticle coupling and assumed that the NRs move in an optically uniform medium (*n* = 1.33). The rotational and translational drag coefficients of an NR were approximated (*37*) by those of a prolate spheroid with a length of 176 nm and a width of 110 nm, which closely mimics the overall shape of an NR (fig. S5A). The resulting translational friction coefficients in water for movement along the spheroid long and short axes were γ_∥_ = 1.165 × 10^−9^ N·s/m and γ_⊥_ = 1.279 × 10^−9^ N·s/m, respectively, while the friction coefficient for rotation around the short axis was γ_rot_ = 8.366 × 10^−24^ Nm·s. Corresponding diffusion constants in the Mie simulations were obtained as *D* = *k*_B_*T*/γ (where *k*_B_ is the Boltzmann constant) for a uniform medium with temperature *T* = 293 K. Further details of the computational methodology can be found in the Supplementary Materials.

## Supplementary Materials

### This PDF file includes:

Supplementary Text Figs. S1 to S13 Legends for movies S1 to S7 References
