# Supplementary material for: Synchronization of optically self-assembled nanorotors
Source: Sci Adv. 2024 Mar 8;10(10):eadn3485. doi: 10.1126/sciadv.adn3485 (PMC10923511; doi:10.1126/sciadv.adn3485)
Supplement: Supplementary file 1 — Supplementary Text Figs. S1 to S13 Legends for movies S1 to S7 References [file sciadv.adn3485_sm.pdf]

Science Advances
AAAS

Supplementary Materials for

Synchronization of optically self-assembled nanorotors

Ximin Cui *et al.*

Corresponding author: Mikael Käll, mikael.kall@chalmers.se

Sci. Adv. **10**, eadn3485 (2024)
DOI: 10.1126/sciadv.adn3485

The PDF file includes:

Supplementary Text
Figs. S1 to S13
Legends for movies S1 to S7
References

Other Supplementary Material for this manuscript includes the following:

Movies S1 to S7

Supplementary Text

Optical forces calculation

The calculation of the optical forces acting on the nanoparticles is a basic ingredient in our simulation of the nanoparticle motion. The electromagnetic fields have been calculated using the Ansys Lumerical FDTD program as well as using T-matrix methods combined with extended Mie theory (*48*, *49*).

Once we know the electric and magnetic fields $\vec{E}$ and $\vec{H}$ on a surface S enclosing a particle, we can calculate the force $\vec{F}$ on the particle and the torque $\vec{M}$ about its center, $\vec{r}_0$, in terms of surface integrals involving the Maxwell stress tensor $\overleftrightarrow{\sigma}$ through (here and in the following $\Re$ denotes the real part)

$$\vec{F} = \frac{1}{2} \oiint \Re[\overleftrightarrow{\sigma} \cdot \hat{n}] dS\,, \tag{1}$$

and

$$\vec{M} = \frac{1}{2} \oiint \Re[(\vec{r} - \vec{r}_0) \times (\overleftrightarrow{\sigma} \cdot \hat{n})] dS\,. \tag{2}$$

The integration, and hence the point $\vec{r}$ and the corresponding outward-pointing normal vector $\hat{n}$, runs over a surface enclosing the particle, see for example (*50*) for further details. The elements of the stress tensor are given by

$$\sigma_{ij} = \varepsilon_0 \varepsilon_r E_i^* E_j + \mu_0 H_i^* H_j - \frac{1}{2} \delta_{ij} \left(\varepsilon_0 \varepsilon_r \left|\vec{E}\right|^2 + \mu_0 \left|\vec{H}\right|^2 \right), \tag{3}$$

where the indices i and j denote the Cartesian coordinate directions and ε_r is the relative permittivity of the medium surrounding the particle (water in our case). We assume that the medium surrounding the particles is non-magnetic.

Simulation of the particle dynamics

With the knowledge of the optical forces we use them in a simulation of the particle motion. In these calculations we model the particles as prolate spheroids with a semiaxis of length c along its cylindrical symmetry axis and a semiaxis a in the perpendicular direction. Their translational motion is restricted to a plane while they can also rotate around one axis that is orthogonal to that plane and to the symmetry axis of the particle. The translational motion follows Newton's second law,

$$m_p \frac{d\vec{v}_i}{dt} + \overleftrightarrow{\gamma}_i \vec{v}_i = \vec{F}_{\text{opt}}^{(i)}(\{\vec{r}\}, \{\varphi\}) + \vec{F}_{\text{st}}^{(i)}, \tag{4}$$

where, however, the first inertial term, involving the particle mass m_p can be neglected.

The second term on the left hand side describes a viscous friction force that varies linearly with the velocity $\vec{v}_i$ of particle i (given that the flow around the particle is associated with a very low Reynolds number). The frictional forces are calculated using the formulas obtained by Perrin (*51*) a long time ago to describe the viscous friction of spheroids and ellipsoids in a liquid. On the right hand side $\vec{F}_{\text{opt}}^{(i)}$ is the optical force, depending on the positions $\{\vec{r}\} = (\vec{r}_1, \vec{r}_2, \ldots)$ and orientations $\{\vec{\varphi}\} = (\vec{\varphi}_1, \vec{\varphi}_2, \ldots)$ of all the particles. The last term on the right hand side of Eq. (4) yields a stochastic force due to thermal fluctuations.

In addition to causing friction and giving rise to stochastic forces, the water surrounding the particles can mediate hydrodynamic interactions between them. As one particle spins around its own axis, it sets water in motion generating a flow at the other particles. However, the speed of the flow decays as $1/r^2$ with increasing distance r from the spinning particle (and the decay is even faster than that in case there is a fixed substrate nearby).

This means that in our case the velocity of the flowing water decreases by at least two orders of magnitude going from the surface of a spinning particle to a neighboring particle. Given the linear relation between torques and spinning frequencies, this also gives us an estimate of the ratio between the torque caused by hydrodynamic interactions and the optical torque. In view of this we conclude that hydrodynamic interactions have very little impact on the rotational motion of the nanoparticles since they only account for torques that are of the order of 1% or less of the optical torques.

In our dynamic simulation then for each time step (typically 2 μs) we calculate a deterministic velocity as a result of the balance between friction and optical forces,

$$\vec{v}_i(t_n) = \overleftrightarrow{\gamma}_i^{-1} \vec{F}_{\text{opt}}^{(i)}, \tag{5}$$

which results in a deterministic displacement during the time-step. To this we add a stochastic displacement ($\Delta_{\|}$ and $\Delta_{\perp}$) drawn from a Gaussian distribution set by the temperature and the diffusion coefficients (the diffusion rates along and orthogonal to the particle symmetry axis differ). Then, the new coordinates for a particle at time $t_{n+1} = t_n + \delta t$ are given by

$$r_{\|}(t_{n+1}) = r_{\|}(t_n) + v_{\|}(t_n)\delta t + \Delta_{\|}, \tag{6}$$

and

$$r_{\perp}(t_{n+1}) = r_{\perp}(t_n) + v_{\perp}(t_n)\delta t + \Delta_{\perp}. \tag{7}$$

The rotational motion is governed by completely analogous equations with the deterministic angular velocity given by

$$\dot{\varphi}_i(t_n) = \gamma_{\text{rot}}^{-1} M_{\text{opt}}^{(i)}. \tag{8}$$

The theoretical heat maps showing the correlations between the scattered light intensity from different particles were calculated using the dynamic simulations based on Mie theory. In these simulations we mimicked the experimental illumination and subsequent scattering of light (figs. S2 and S7) by following the orientation of the particles during time intervals of length $T_f = 100$ μs. In the simulation, these time intervals follow each other without intermissions. Given that the scattering of the probing light is primarily caused by dipolar radiation emanating from the long axis dipole of the particles, to a first approximation the scattered electric field scales as $cos\varphi_i$, and the scattered intensity, apart from a constant, is proportional to $cos^2\varphi_i$, see fig. S7, and we will from now on refer to it as normalized intensity. Hence, to calculate the variations in scattering intensity we run the dynamic simulation using a time step of 2 μs. At each time step the orientation of each particle is recorded and then the "flash" average of the normalized intensity

$$\langle cos^2\varphi_i \rangle(t_0) = T_f^{-1} \int_{t_0}^{t_0+T_f} cos^2\varphi_i(t)dt\,, \tag{9}$$

is calculated for each particle and each (theoretical) flash. The so obtained heat maps of normalized intensity show a clear correlation between different particles, provided the incident laser power that causes optical binding and drives the rotational motion is sufficiently strong. In simple terms this means that the different nanoparticles tend to have their major axis' oriented in nearby directions, an effect caused by the electromagnetic coupling between them.

To get a quantitative measure of the synchronization of the spinning motion we have calculated the order parameter (here N_p is the total number of particles)

$$r(t) = \frac{1}{N_p}\left|\sum_{i=1}^{N_p} e^{2i\varphi_i(t)}\right|, \tag{10}$$

and averaged it in time over the course of the simulations

$$\langle r \rangle = \frac{1}{N_T}\sum_{n=1}^{N_T} r(t_n)\,, \tag{11}$$

with up to $N_T = 2.5 \times 10^6$ points in time. The results show values of $\langle r \rangle$ ranging from 0.81 to 0.91 for the laser powers used in the case of two spinning particles.

Qualitative discussion of the synchronization mechanism

The basic mechanism behind the synchronization of the particles' spinning can be understood within a simple model involving electric dipole-dipole interactions. Similar models have been applied in the analysis of other experiments involving optical interactions and synchronization (*24, 31*).

Consider two point dipoles situated in a plane at a distance d from each other, as illustrated in fig. S6A, with the dimer axis aligned with the *x* axis. We assume that the dipoles are immersed in water and that typically the distance between them equals the optical wavelength in water λ_0/n for the incident light driving the dipoles. Assume also, for the moment, that the dipoles are highly anisotropic with a considerable polarizability only in one direction in the plane of the dimer (that is the *xy* plane).

These dipoles are now forced to spin by an external electric field,

$$\vec{E}_0 = \hat{x}E_{0x} + \hat{y}E_{0y}, \tag{12}$$

equal at both dipoles, and with an implicit time dependence $e^{-i\omega t}$. The response of the dipoles are determined by an effective polarizability tensor $\overleftrightarrow{\alpha}_i$ yielding

$$\vec{p}_i = \overleftrightarrow{\alpha}_i \vec{E}_i, \tag{13}$$

where i denotes the particle number. Note that $\vec{E}_i$ in the above equation denotes the total field at dipole i, also including fields that have been scattered off the other dipole.

Given the highly anisotropic response of the dipole the polarizability tensor depends on the angle φ_i formed between the dipole axis and the dimer axis, hence

$$\overleftrightarrow{\alpha}_i = \frac{\alpha_0}{2}\begin{bmatrix} 1 + cos2\varphi_i & sin2\varphi_i \\ sin2\varphi_i & 1 - cos2\varphi_i \end{bmatrix}, \tag{14}$$

where α_0 is a constant with real and imaginary parts α_0' and α_0'' respectively,

$$\alpha_0 = \alpha_0' + i\alpha_0''. \tag{15}$$

The imaginary part is due to losses, partly through absorption but also, since this is an effective polarizability, as a result of scattering.

The time-averaged (over one period of the electromagnetic oscillation) torque on particle (dipole) 1 can be determined from the simple expression ($\Re$ and $\Im$ denote the real and imaginary parts, respectively)

$$M_{1z} = \frac{1}{2}\Re\left[\hat{z}\cdot\left(\vec{p}_1^{\,*} \times \vec{E}_1\right)\right], \tag{16}$$

which can be written as

$$M_{1z} = \frac{\alpha_0'}{4}\left[\left(\left|E_{1y}\right|^2 - \left|E_{1x}\right|^2\right) sin2\varphi_1 + 2\Re\left(E_{1x}^* E_{1y}\right) cos2\varphi_1\right] + \frac{\alpha_0''}{4}\left[2\Im\left(E_{1x}^* E_{1y}\right)\right]. \tag{17}$$

With one single dipole, the electric fields entering this expression are identical with the incident field $\vec{E}_0$. Linearly polarized light will then align the dipole along the direction of the incident field. For circularly polarized light, on the other hand, the first part of the torque in Eq. (17), associated with the real part of the polarizability, vanishes, whereas the second part, proportional to α_0'', yields a torque that turns the particle in the direction of the circular polarization.

Next we look specifically at the dipole-dipole interaction. With a separation between the dipoles equal to the wavelength of light in the medium, this interaction is approximately limited to dipole moments perpendicular to the dimer axis (dipole in the *y* direction) giving rise to a field in the same direction on the other dipole. That is, the total field at dipole 1 can approximately be written

$$\vec{E}_1 = \hat{x}E_{0x} + \hat{y}E_{0y} + \delta\vec{E}_1 \approx \hat{x}E_{0x} + \hat{y}E_{0y} + \hat{y}\kappa p_{2y}. \tag{18}$$

In the particular situation we are considering κ is, to a good approximation, real and positive and in the following we treat it as a real and positive constant. We further take the incident field to be $\vec{E}_0 = E_0(\hat{x} + i\hat{y})$. Then the correction $\delta\vec{E}_1$ to the field causes a first-order correction (in κ) to the optical torque equal to

$$\delta M_{1z} = \frac{\kappa}{4}|E_0|^2\{\alpha_0'^2[sin2\varphi_1 + sin(2\varphi_2 - 2\varphi_1)] + \alpha_0'\alpha_0''[1 + cos(2\varphi_2 - 2\varphi_1) - cos2\varphi_1 - cos2\varphi_2] + \alpha_0''^2 sin2\varphi_2\}. \tag{19}$$

In the cases we consider here, the real part of the polarizability dominates the imaginary part which means that the leading corrections to the torque caused by particle-particle interactions comes from the first two terms, proportional to $\alpha_0'^2$, in Eq. (19).

The first term in δM_{1z}, varying as $sin2\varphi_1$ (but still related to interactions as it is proportional to κ), tends to align the dipole with the *y* direction. This is due to the fact that there is an additional electric field in this direction (which is in phase with the *y* component of the external field). This contribution to the torque will in general cause the dipole to spend more time pointing in the *y* direction than the *x* direction while performing its spinning motion. As a result of this phenomenon we see oscillations in the spinning frequencies in the simulations.

The second term in Eq. (19), varying as $sin(2\varphi_2 - 2\varphi_1)$, is even more interesting. We see that if the two angles φ_1 and φ_2 differ, the case with a somewhat larger φ_2 will give an additional positive contribution to the torque M_{1z} that means that φ_1 will tend to catch up with φ_2, and vice versa. This interaction, which is identical in form to the interaction causing synchronization in the Kuramoto model (*38*) is at the core of the synchronization of the spinning motion of two or more nanoparticles that we see in our experiments and simulations. Moreover, the order parameter defined in Eq. (10) is also used as a measure of synchronization in the Kuramoto model.

To make contact with the expressions for the spinning frequencies given in the main text, and the quantities f_0 and Δf used there, we have

$$f_0 = (2\pi)^{-1}\alpha_0''|E_0|^2/(2\gamma_{\rm rot}), \tag{20}$$

and

$$\Delta f = (2\pi)^{-1}\kappa\alpha_0'^2|E_0|^2/(4\gamma_{\rm rot}). \tag{21}$$

To check the validity of the above reasoning we have, using the T-matrix formalism, calculated the torques on two gold particles in the form of prolate spheroids with semi-axis of 55 nm and 88 nm, respectively. Figure S6B shows calculated results for the torque in three different scenarios (i) the particle response is limited to the electric dipole along the major axis, (ii) the response is limited to electric dipole response along all three axes, and (iii) we allow for the full response of the particle including all relevant multipoles. For comparison a fourth curve shows the

(orientation-independent) torque one would get if the particles were spheres with a radius of 88 nm.

The results show that the assumption of a particle that can only be polarized along one direction is too simple in the sense that it overestimates the torque as well as the corrections to the torque due to particle-particle interactions. Allowing for electric dipole response along all axes, on the other hand, give results in close agreement with those obtained including the full response of the particles.

Figure S6C shows the torque on particle 1 as a function of the orientations of both particles, φ_1 and φ_2. These results, to a large degree, confirm the qualitative predictions of the dipole model above. The torque M_{1z} shows a strong variation with φ_1 similar to the first term in Eq. (19) and also a variation with $\varphi_2 - \varphi_1$ similar to the second term in Eq. (19).

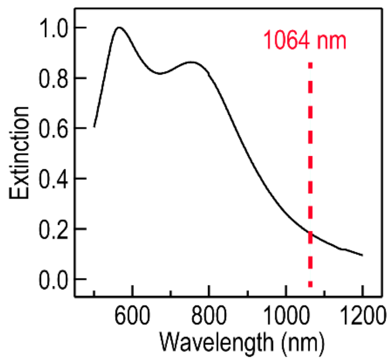

Fig. S1.

Optical properties. Measured ensemble-averaged extinction spectrum of the gold nanorod (Au NR) sample. The red dash line indicates the trapping wavelength of 1064 nm.

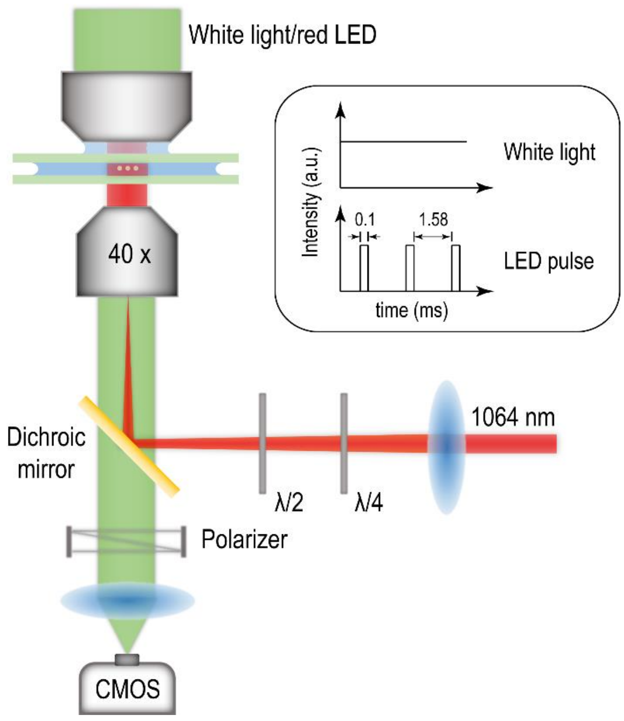

Fig. S2.

Optical setup. Schematic of the optical setup used to observe self-organization and synchronization of NR optical matter arrays. The NRs are trapped in two dimensions against the upper cover glass of thin a liquid sample cell by a 1064 nm wavelength laser beam. The beam size was truncated by an iris before being focused on the back-focal plane of the objective in order to minimize beam divergence at the sample plane. The polarization of the beam was adjusted to left circular by manually tuning a half-wave and a quarter-wave plate. The NRs are illuminated through an oil-immersion dark-field (DF) condenser with white light from the microscope halogen lamp or red light from a LED. For stroboscopic observation of NR rotation behavior, a linear polarizer is inserted in front of the CMOS camera and the on/off time of the LED is set to 0.1/1.58 ms while the camera frame rate is set to 633 Hz.

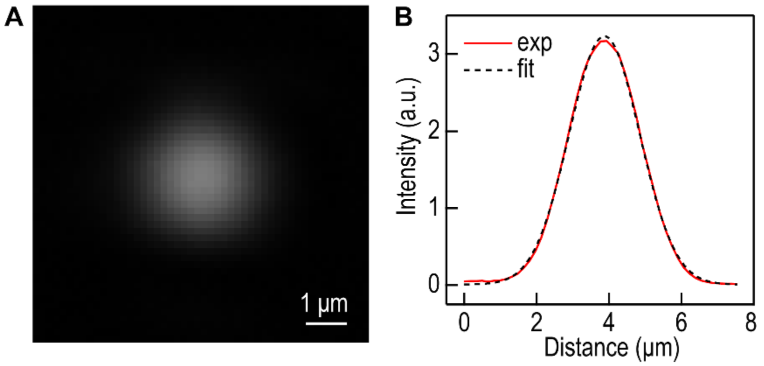

Fig. S3.

Trapping laser focus. (**A**) Optical image of the laser spot captured by placing a mirror at the trapping plane. (**B**) Measured (red line) and Gaussian fit (black dashed line) of the intensity profile in the trapping plane. The FWHM of the profile is ~2.5 μm.

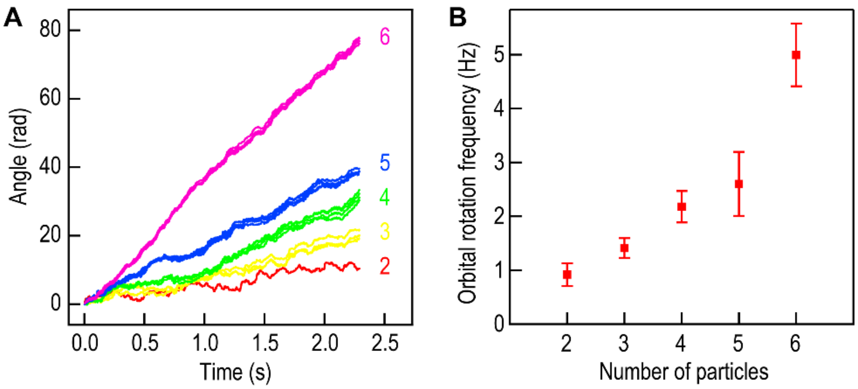

Fig. S4.

Orbital rotation characteristics. (**A**) Evolution of NR angle relative to *x*-axis for arrays of 2-6 close-packed NRs. (**B**) Orbital frequency versus number of NRs from the data in (**A**). The incident laser power was ~430 mW.

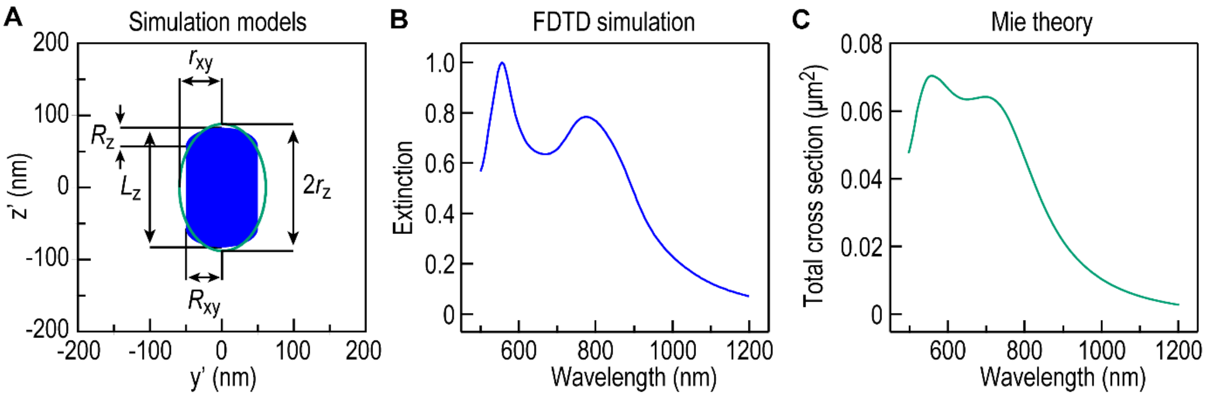

Fig. S5.

Model NRs. (**A**) NR dimensions used in simulations. FDTD simulations were based on modelling a NR as a cylinder with ellipsoidal caps with R_{xy} = 50 nm, R_z = 26 nm, and L_z = 166 nm (blue). Mie calculations and Brownian diffusion simulations were based on modelling a NR as a prolate spheroid with minor axis r_{xy} = 55 nm and major axis r_z = 88 nm (green). (**B**) Simulated extinction spectrum of a NR using FDTD. (**C**) Calculated orientation-averaged extinction spectrum of NRs using Mie theory.

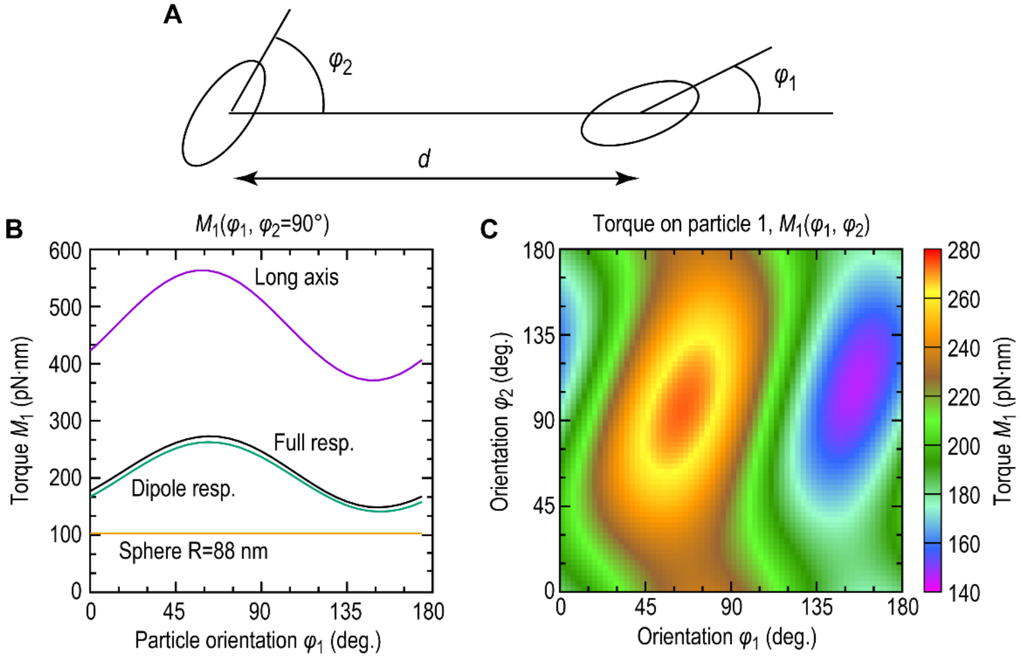

Fig. S6.

Variation in optical torque for a dimer of prolate spheroids. (**A**) Schematic illustration of the particles forming the dimer and their orientations. (**B**) Calculated torque M_1 on a prolate spheroidal nanoparticle with semi-axis 55 nm and 88 nm, respectively. The particle is separated from another identical particle by a distance of 810 nm and both particles are illuminated by a plane electromagnetic wave at normal incidence with a wavelength of 1064 nm and intensity 0.1W/(μm^2).

Here the other particle has a fixed orientation with its long axis perpendicular to the dimer axis. (**C**) Color plot of the torque M_1 as a function of the orientation of both particles in the dimer.

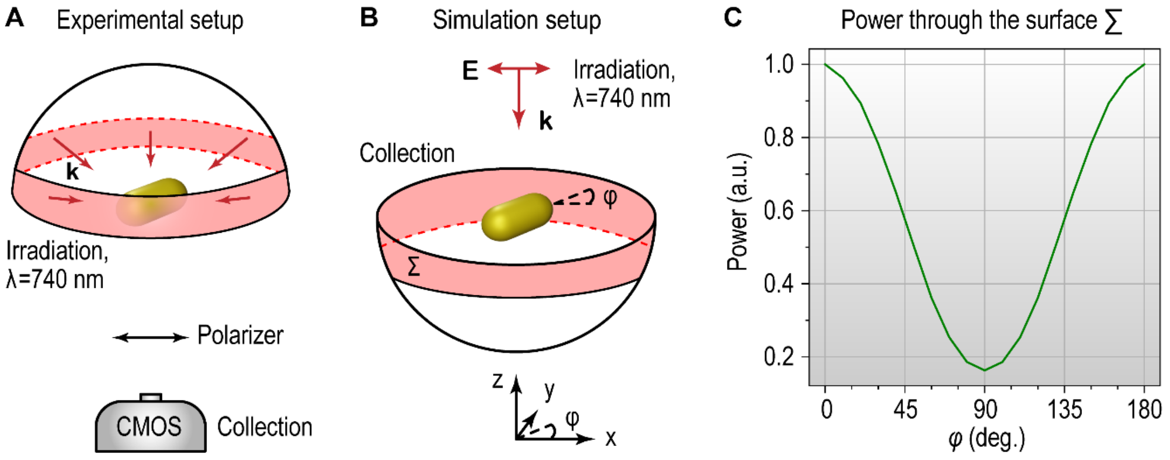

Fig. S7.

Methodology to determine NRs' orientations. (**A**) Schematics of the experiment. The sample is illuminated through a DF oil condenser with NA=1.2–1.43 using unpolarized red light (λ=740 nm) from an LED. The refractive indexes of the used oil, cover glass and water are 1.515, 1.52 and 1.33, respectively. Light scattered in the forward direction pass through a linear polarizer oriented along the *x*-axis and is collected by a CMOS camera. (**B**) Simulated DF intensity versus NR orientation. The NR is illuminated with an x-polarized plane wave of wavelength λ=740 nm. The power scattered into the forward angular range between 64.5 and 90° light is analyzed as a function of angle φ between the NR long axis and the *x*-axis. Due to electromagnetic reciprocity, this set up is a good representation of the experiment. (**C**) Simulated DF intensity versus NR angle φ.

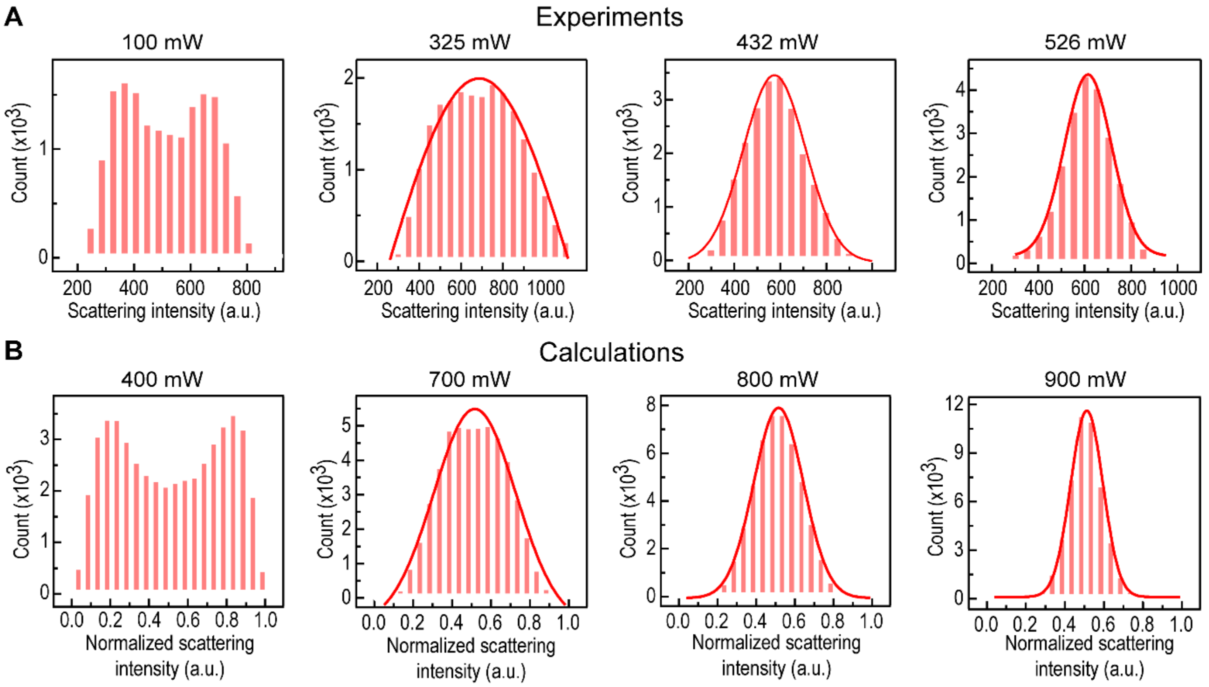

Fig. S8.

Laser power-dependent scattering intensity distributions. (**A**) Measured distribution of single particle scattering intensities recorded using stroboscopic illumination of optically bound NR dimers trapped using ~100, 320, 430, and 530 mW laser power, respectively. (**B**) Calculated distribution of single particle scattering intensities by running a dynamic simulation with two optically bound prolate spheroids using 400, 700, 800, and 900 mW laser power, respectively.

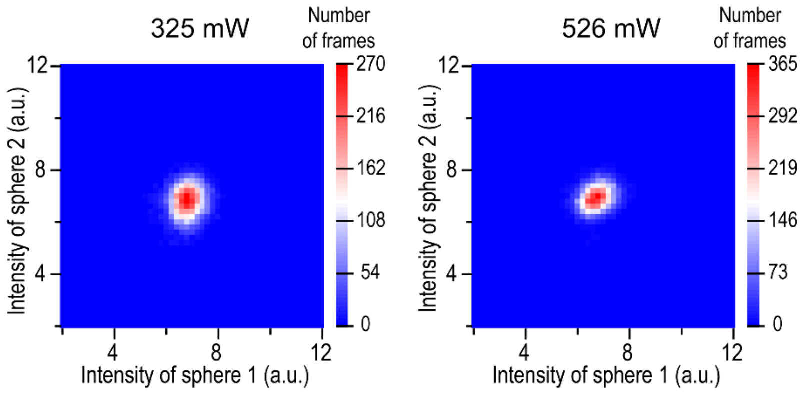

Fig. S9.

Intensity statistics for dimers of optically bound Au nanospheres. Scatter plots of intensities I_1 vs. I_2, extracted from >10000 separate images of two pairs of nominally spherical Au nanoparticles and binned into heat-maps, for two different laser powers. The distributions are narrow and almost circular.

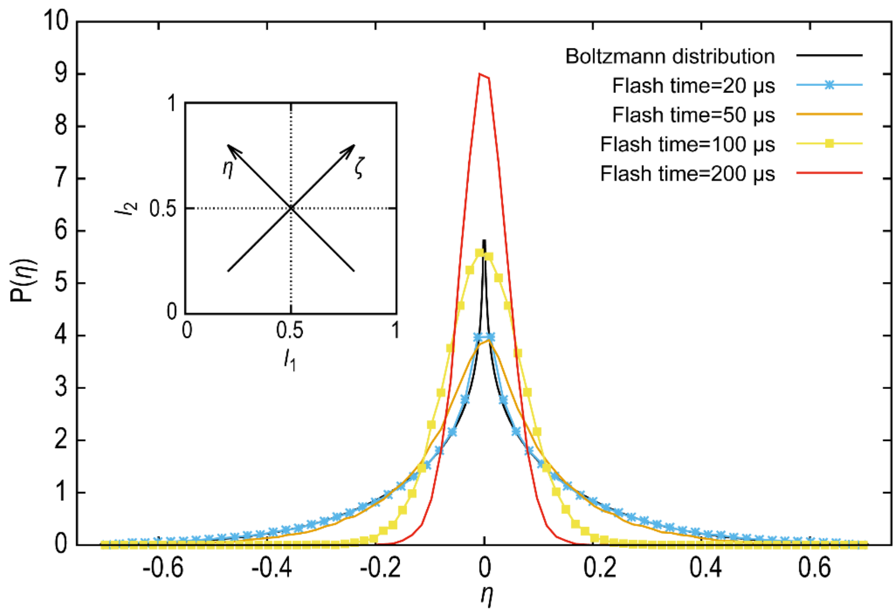

Fig. S10.

Simulated intensity distribution vs. flash time. The figure shows the cumulated intensity probability distribution perpendicular to the diagonal I_1=I_2, i.e. across the heat map ridge, from stochastic simulations of an optically bound dimer versus simulated integration times ("flash-time"). The shortest flash-time produces a distribution with a sharp cusp at $\eta = 0$. This is consistent with a Boltzmann distribution in angular space (φ_1, φ_2) based on the nanorod interaction.

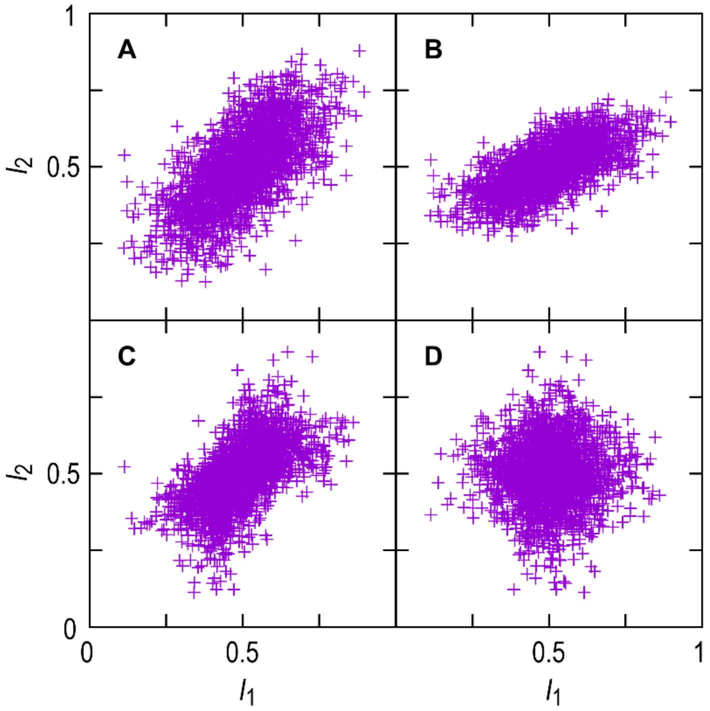

Fig. S11.

Simulated intensity distribution and randomization tests. The figure shows simulated intensity correlations I_1 vs. I_2 from stochastic simulations of an optically bound NR dimer. (**A**) Original data; (**B**) Result when I_1 is reduced by a factor 0.6; (**C**) Result when I_1 is reduced by a factor 0.6 and the particle identities are scrambled; (**D**) Result when I_1 is reduced by a factor 0.6, the I_2 data is delayed by 1 s to cancel all correlations, and the particle identities are scrambled. The data shows that the positive co-variance is only marginally affected by a moderate variation in particle scattering amplitude and by scrambling the particle identities (as in the experiments).

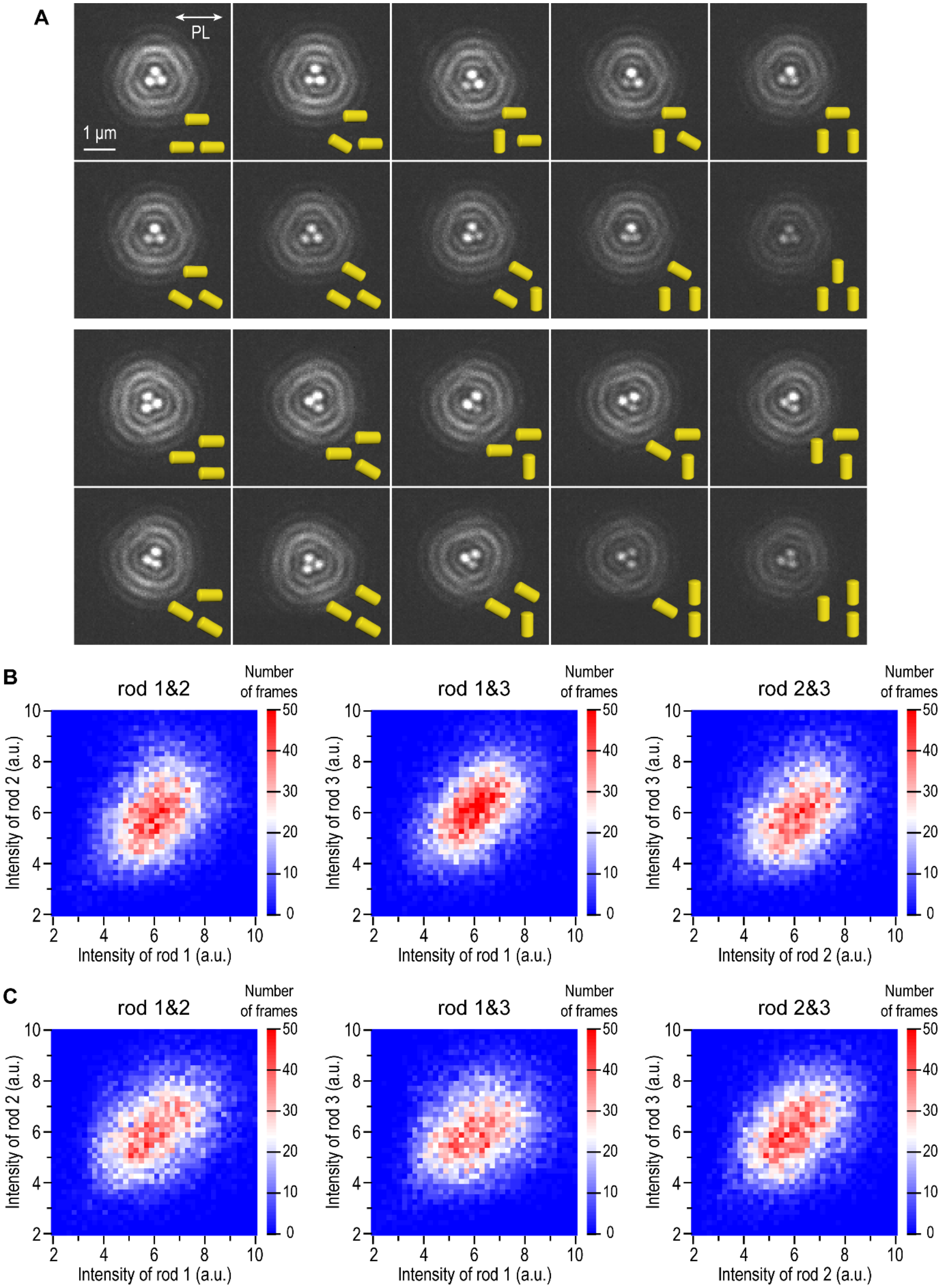

A
PL
1 μm
B
rod 1&2
Number of frames
Intensity of rod 1 (a.u.)
Intensity of rod 2 (a.u.)
rod 1&3
Intensity of rod 3 (a.u.)
rod 2&3
C
rod 1&2
rod 1&3
rod 2&3

Fig. S12.

Statistical analyses of two additional NR trimers. (**A**) DF microscopy using stroboscopic red-light illumination combined with polarization-filtered imaging reveal intensity fluctuations due to variations in NR orientation with the respect to the polarizer. Snapshots of a trimer using stroboscopic red-light illumination combined with polarization-filtered imaging together with schematics indicating possible NR orientations compatible with the recorded particle intensities. (**B, C**) Grid heat maps of the intensity correlations for two additional trimers measured using an applied laser power of ~430 mW.

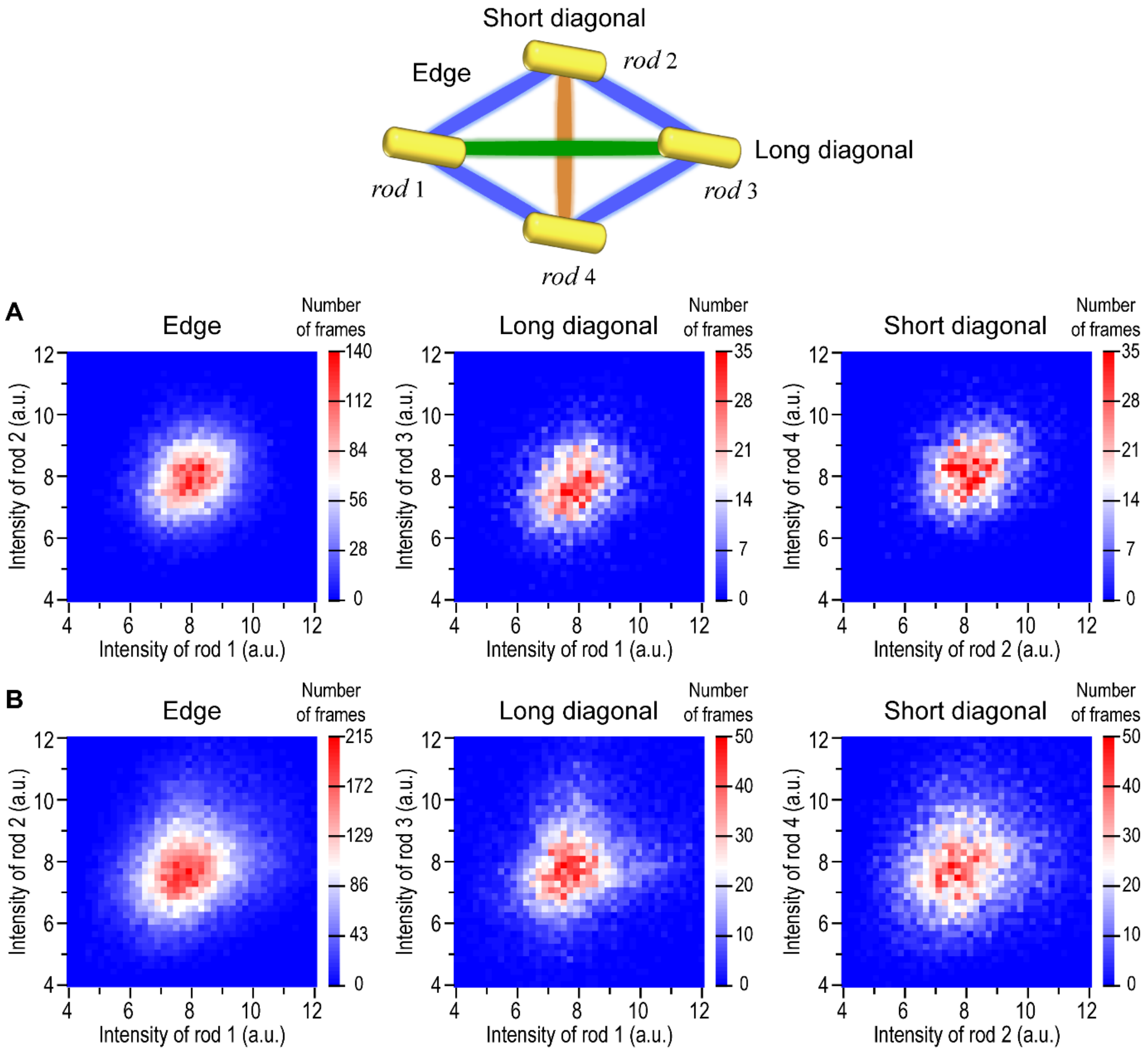

Fig. S13.

Statistical analyses of two additional NR tetramers. (**A, B**) Grid heat maps of the intensity correlations for two additional trimers measured using an applied laser power of ~430 mW.

Movie S1.
Random transitions of three optically bound gold nanorods.

Movie S2.
Random transitions of four optically bound gold nanorods.

Movie S3.
Random transitions of five optically bound gold nanorods.

Movie S4.
Orbital rotation of two optically bound gold nanorods.

Movie S5.
Orbital rotation of three optically bound gold nanorods.

Movie S6.
Orbital rotation of four optically bound gold nanorods.

Movie S7.
Orbital rotation of five optically bound gold nanorods.

Movie S1 to S3 have been slowed down by a factor 100.
Movie S4 to S7 have been slowed down by a factor 10.

REFERENCES AND NOTES

1. C. Huygens, *Horologium Oscillatorium* (The Pendulum Clock) (Apud F. Muguet, 1673).

2. F. K. Lamb, J. J. Aly, M. Cook, D. Q. Lamb, Synchronization of magnetic stars in binary systems, *Astrophys. J.* **274**, L71–L75 (1983).

3. M. Fussenegger, Synchronized bacterial clocks, *Nature* **463**, 301–302 (2010).

4. A. F. Taylor, M. R. Tinsley, F. Wang, Z. Y. Huang, K. Showalter, Dynamical quorum sensing and synchronization in large populations of chemical oscillators, *Science* **323**, 614–617 (2009).

5. H. Y. Lee, H.-W. Lee, D. Kim, Origin of synchronized traffic flow on highways and its dynamic phase transitions, *Phys. Rev. Lett.* **81**, 1130–1133 (1998).

6. M. Zahedinejad, H. Fulara, R. Khymyn, A. Houshang, M. Dvornik, S. Fukami, S. Kanai, H. Ohno, J. Åkerman, Memristive control of mutual spin Hall nano-oscillator synchronization for neuromorphic computing, *Nat. Mater.* **21**, 81–87 (2022).

7. J.-D. Deschênes, L. C. Sinclair, F. R. Giorgetta, W. C. Swann, E. Baumann, H. Bergeron, M. Cermak, I. Coddington, N. R. Newbury, Synchronization of distant optical clocks at the femtosecond level, *Phys. Rev. X* **6**, 021016 (2016).

8. J. Li, Z.-H. Zhou, S. Wan, Y. L. Zhang, Z. Shen, M. Li, C.-L. Zou, G.-C. Guo, C.-H. Dong, All-Optical synchronization of remote optomechanical systems, *Phys. Rev. Lett.* **129**, 063605 (2022).

9. H. Bergeron, L. C. Sinclair, W. C. Swann, I. Khader, K. C. Cossel, M. Cermak, J.-D. Deschênes, N. R. Newbury, Femtosecond time synchronization of optical clocks off of a flying quadcopter, *Nat. Commun.* **10**, 1819 (2019).

10. A. Pikovsky, M. Rosenblum, J. Kurths, *Synchronization: A Universal Concept in Nonlinear Sciences* (Cambridge Univ. Press, 2003).

11. K. Han, G. Kokot, S. Das, R. G. Winkler, G. Gompper, A. Snezhko, Reconfigurable structure and tunable transport in synchronized active spinner materials, *Sci. Adv.* **6**, eaaz8535 (2020).

12. S. G. Leyva, R. L. Stoop, I. Pagonabarraga, P. Tierno, Hydrodynamic synchronization and clustering in ratcheting colloidal matter, *Sci. Adv.* **8**, eabo4546 (2022).

13. A. Ashkin, J. M. Dziedzic, J. E. Bjorkholm, Steven. Observation of a single-beam gradient force optical trap for dielectric particles, *Opt. Lett.* **11**, 288–290 (1986).

14. D. G. Grier, A revolution in optical manipulation. *Nature* **424**, 810–816 (2003).

15. M. M. Burns, J.-M. Fournier, J. A. Golovchenko, Optical binding. *Phys. Rev. Lett.* **63**, 1233–1236 (1989).

16. M. M. Burns, J.-M. Fournier, J. A. Golovchenko, Optical matter: Crystallization and binding in intense optical fields. *Science* **249**, 749–754 (1990).

17. H. He, M. E. Friese, N. R. Heckenberg, H. Rubinsztein-Dunlop, Direct observation of transfer of angular momentum to absorptive particles from a laser beam with a phase singularity. *Phys. Rev. Lett.* **75**, 826–829 (1995).

18. M. E. Friese, T. A. Nieminen, N. R. Heckenberg, H. Rubinsztein-Dunlop, Optical alignment and spinning of laser-trapped microscopic particles. *Nature* **394**, 348–350 (1998).

19. L. M. Tong, V. D. Miljković, M. Käll, Alignment, rotation, and spinning of single plasmonic nanoparticles and nanowires using polarization dependent optical forces, *Nano Lett.* **10**, 268–273 (2010).

20. A. Lehmuskero, P. Johansson, H. Rubinsztein-Dunlop, L. M. Tong, M. Käll, Laser trapping of colloidal metal nanoparticles. *ACS Nano* **9**, 3453–3469 (2015).

21. V. Demergis, E.-L. Florin, Ultrastrong optical binding of metallic nanoparticles. *Nano Lett.* **12**, 5756–5760 (2012).

22. F. Han, Z. J. Yan, Phase transition and self-stabilization of light-mediated metal nanoparticle assemblies. *ACS Nano* **14**, 6616–6625 (2020).

23. J. Parker, C. W. Peterson, Y. Yifat, S. A. Rice, Z. J. Yan, S. K. Gray, N. F. Scherer, Optical matter machines: Angular momentum conversion by collective modes in optically bound nanoparticle arrays. *Optica* **7**, 1341–1348 (2020).

24. N. Sule, Y. Yifat, S. K. Gray, N. F. Scherer, Rotation and negative torque in electrodynamically bound nanoparticle dimers. *Nano Lett.* **17**, 6548–6556 (2017).

25. F. Han, J. A. Parker, Y. Yifat, C. Peterson, S. K. Gray, N. F. Scherer, Z. J. Yan, Crossover from positive to negative optical torque in mesoscale optical matter. *Nat. Commun.* **9**, 4897 (2018).

26. F. Nan, X. Li, S. L. Zhang, J. Ng, Z. Yan, Creating stable trapping force and switchable optical torque with tunable phase of light. *Sci. Adv.* **8**, eadd6664 (2022).

27. F. Nan, Z. J. Yan, Light-driven self-healing of nanoparticle-based metamolecules. *Angew. Chem. Int. Ed.* **131**, 4971–4976 (2019).

28. L. Shao, M. Käll, Light-driven rotation of plasmonic nanomotors. *Adv. Funct. Mater.* **28**, 1706272 (2018).

29. L. Shao, Z.-J. Yang, D. Andrén, P. Johansson, M. Käll, Gold nanorod rotary motors driven by resonant light scattering. *ACS Nano* **9**, 12542–12551 (2015).

30. O. Brzobohatý, A. V. Arzola, M. Šiler, L. Chvátal, P. Jákl, S. Simpson, P. Zemánek, Complex rotational dynamics of multiple spheroidal particles in a circularly polarized, dual beam trap, *Opt. Express* **23**, 7273–7287 (2015).

31. S. H. Simpson, L. Chvátal, P. Zemánek, Synchronization of colloidal rotors through angular optical binding, *Phys. Rev. A* **93**, 023842 (2016).

32. O. Brzobohatý, M. Duchaň, P. Jákl, J. Ježek, M. Šiler, P. Zemánek, S. H. Simpson, Synchronization of spin-driven limit cycle oscillators optically levitated in vacuum. *Nat. Commun.* **14**, 5441 (2023).

33. S. H. Simpson, P. Zemánek, O. M. Maragò, P. H. Jones, S. Hanna, Optical binding of nanowires. *Nano Lett.* **17**, 3485–3492 (2017).

34. X. C. Ye, C. Zheng, J. Chen, Y. Z. Gao, C. B. Murray, Using binary surfactant mixtures to simultaneously improve the dimensional tunability and monodispersity in the seeded growth of gold nanorods. *Nano Lett.* **13**, 765–771 (2013).

35. F. Nan, F. Han, N. F. Scherer, Z. J. Yan, Dissipative self-assembly of anisotropic nanoparticle chains with combined electrodynamic and electrostatic interactions. *Adv. Mater.* **30**, e1803238 (2018).

36. Z. J. Yan, S. K. Gray, N. F. Scherer, Potential energy surfaces and reaction pathways for light-mediated self-organization of metal nanoparticle clusters. *Nat. Commun.* **5**, 1038 (2014).

37. F. Perrin, Mouvement brownien d'un ellipsoide - I. Dispersion diélectrique pour des molécules ellipsoidales. *J. Phys. Radium* **5**, 497–511 (1934).

38. J. A. Acebrón, L. L. Bonilla, C. J. Pérez Vicente, F. Ritort, R. Spigler, The Kuramoto model: A simple paradigm for synchronization phenomena. *Rev. Mod. Phys.* **77**, 137–185 (2005).

39. D. Andrén, D. G. Baranov, S. Jones, G. Volpe, R. Verre, M. Käll, Microscopic metavehicles powered and steered by embedded optical metasurfaces. *Nat. Nanotechnol.* **16**, 970–974 (2021).

40. X. F. Wu, R. Ehehalt, G. Razinskas, T. Feichtner, J. Qin, B. Hecht, Light-driven microdrones. *Nat. Nanotechnol.* **17**, 477–484 (2022).

41. L. Shao, D. Andrén, S. Jones, P. Johansson, M. Käll, Optically controlled stochastic jumps of individual gold nanorod rotary motors. *Phys. Rev. B* **98**, 085404 (2018).

42. F. Tebbenjohanns, M. Luisa Mattana, M. Rossi, M. Frimmer, L. Novotny, Quantum control of a nanoparticle optically levitated in cryogenic free space. *Nature* **595**, 378–382 (2021).

43. J. Rieser, M. A. Ciampini, H. Rudolph, N. Kiesel, K. Hornberger, B. A. Stickler, M. Aspelmeyer, U. Delić, Tunable light-induced dipole-dipole interaction between optically levitated nanoparticles. *Science* **377**, 987–990 (2022).

44. R. Reimann, M. Doderer, E. Hebestreit, R. Diehl, M. Frimmer, D. Windey, F. Tebbenjohanns, L. Novotny, GHz rotation of an optically trapped nanoparticle in vacuum. *Phys. Rev. Lett.* **121**, 033602 (2018).

45. F. van der Laan, R. Reimann, M. Doderer, E. Hebestreit, R. Diehl, M. Frimmer, D. Windey, F. Tebbenjohanns, L. Novotny, Erratum: GHz rotation of an optically trapped nanoparticle in vacuum [Phys. Rev. Lett. 121, 033602 (2018)]. *Phys. Rev. Lett.* **126**, 159901 (2021).

46. B. A. Stickler, K. Hornberger, M. S. Kim, Quantum rotations of nanoparticles. *Nat. Rev. Phys.* **3**, 589–597 (2021).

47. J.-Y. Tinevez, N. Perry, J. Schindelin, G. M. Hoopes, G. D. Reynolds, E. Laplantine, S. Y. Bednarek, S. L. Shorte, K. W. Eliceiri, TrackMate: An open and extensible platform for single particle tracking. *Methods* **115**, 80–90 (2017).

48. P. C. Waterman, Symmetry, unitarity, and geometry in electromagnetic scattering. *Phys. Rev. D* **3**, 825–839 (1971).

49. B. Peterson, S. Ström, TMatrix for electromagnetic scattering from an arbitrary number of scatterers and representations of E(3). *Phys. Rev. D* **8**, 3661–3678 (1973).

50. J. P. Barton, D. R. Alexander, S. A. Schaub, Theoretical determination of net radiation force and torque for a spherical particle illuminated by a focused laser beam. *J. Appl. Phys.* **66**, 4594–4602 (1989).
